# Observation and quantification of the morphological effect of trypan blue rupturing dead or dying cells

**DOI:** 10.1371/journal.pone.0227950

**Published:** 2020-01-24

**Authors:** Leo Li-Ying Chan, William L. Rice, Jean Qiu

**Affiliations:** Department of Advanced Technology R&D, Nexcelom Bioscience LLC., Lawrence, Massachusetts, United States of America; The Ohio State University, UNITED STATES

## Abstract

Trypan blue has long been the gold standard for staining dead cell to determine cell viability. The dye is excluded from membrane-intact live cells, but can enter and concentrate in membrane-compromised dead cells, rendering the cells dark blue. Over the years, there has been an understanding that trypan blue is inaccurate for cell viability under 80% without scientific support. We previously showed that trypan blue can alter the morphology of dead cells to a diffuse shape, which can lead to over-estimation of viability. Here, we investigate the origin of the dim and diffuse objects after trypan blue staining. Utilizing image and video acquisition, we show real-time transformation of cells into diffuse objects when stained with trypan blue. The same phenomenon was not observed when staining cells with propidium iodide. We also demonstrate the co-localization of trypan blue and propidium iodide, confirming these diffuse objects as cells that contain nuclei. The videos clearly show immediate cell rupturing after trypan blue contact. The formation of these diffuse objects was monitored and counted over time as cells die outside of the incubator. We hypothesize and demonstrate that rapid water influx may have caused the cells to rupture and disappear. Since some dead cells disappear after trypan blue staining, the total can be under-counted, leading to over-estimation of cell viability. This inaccuracy could affect the outcomes of cellular therapies, which require accurate measurements of immune cells that will be infused back into patients.

## Introduction

Cell viability measurement is one of the most important characterizations for cellular therapy. It is critical to accurately measure the viability for immune cell samples that will be re-introduced into patients. Inaccurate cell viability measurement may lead to inefficacy or induce unwanted autoimmune responses in patients undergoing therapeutic treatments [1–3]. The U.S. Congress has also recognized the importance of cell counting and cell viability measurement standards in the 21^st^ Century Cure Act for cell and gene therapy. Cell counting and viability measurement assurance were identified as opportunity for standards development in the workshop, “Synergizing Efforts in Standards Development for Cellular Therapies and Regenerative Medicine Products”, held by the U.S. Food and Drug Administration (FDA). Under the consultation of National Institute of Standards and Technology (NIST) and other stakeholders, various cell counting and cell viability measurement assurance tools were investigated for improving the quality of cell therapy products [4].

For cell viability measurement, the trypan blue (TB) dye has been used for over a century [5,6]. Although many issues have been documented, such as protein aggregation [7–9], a limited counting time window [10], and inaccurate measurement when viability is less than 80% [11–13], TB remains the go-to viability dye. TB is an azo dye that has a molecular weight of 960 Da [6,14]; it can concentrate in membrane-compromised dead cells, but is excluded from membrane-impermeable live cells [15,16]. We previously demonstrated that TB can cause morphological changes in dead or dying cell populations (forming dim and diffuse objects), and we quantitatively showed that TB assays over-estimate viability when experimental samples fall below 80% viability [13].

Our previous study mused on the identity of the diffuse objects in a TB-stained cell sample [13]. Live (bright) and dead (dark, tight) cells are typically counted manually using a hemacytometer or automatically using a bright field cell counter. However, the diffuse TB-positive objects are only faintly visible under a light microscope (S1 Fig) and are therefore often not counted, potentially leading to inaccurate viability measurements. There is often a 10 to 15% viability difference between TB and nuclear fluorescent viability stains, with the latter method yielding lower viability measurements.

In this work, we investigate the formation of these dim and diffuse objects in TB-stained Jurkat cell samples in videos capturing the staining process in real time. Utilizing propidium iodide (PI), a fluorescent viability dye that stains the nuclei of membrane-compromised dead cells, we show that these diffuse objects are PI-positive, confirming that these are dead cells. The videos show morphological changes occur in dead cells immediately after contact with TB. Specifically, TB-positive cells expand and rupture, leading to the diffuse morphology. In contrast, PI staining neither induces morphological changes nor ruptures the cell body. In addition, as the cells die in an unfavorable environment, the diffuse objects increase over time. The visual evidence recorded in the experiments reveal that TB can induce the formation of these diffuse objects and ultimately leads to over-estimation of viability, which may have significant effects for downstream cellular therapy assays.

## Materials and methods

### Cell culture and reagent preparation

The Jurkat cell line (TIB-152, ATCC, Manassas, VA) was cultured in RPMI 1640 (Gibco, Gaithersburg, MD) supplemented with 10% FBS (Gibco) and 1% penicillin/streptomycin (Sigma-Aldrich, St. Louis, MO). The Jurkat cell culture was maintained in an incubator at 37°C with 5% CO_2_. TB was purchased from BioVision (San Francisco, CA) and used at 0.4%, 0.2%, and 0.1% working concentration. PI was obtained from Nexcelom Bioscience (Lawrence, MA) and used directly from the bottle.

### Cellometer image cytometry instruments and disposable counting chamber

The Cellometer image cytometry instruments (AutoT4, AutoM10, Vision 5X/10X) were employed to capture images and videos of the morphological effects of TB and PI on dead cells [13]. Cellometer AutoT4 and AutoM10 are bright field-only automated cell counters with 4 and 10X optical objectives and a color camera for imaging and video acquisition. Cellometer Vision are bright field and fluorescent image cytometers with a 5X or 10X objective. They use a monochromatic camera with two fluorescent channels: green (EX: 475 nm, EM: 527 nm) and red (EX: 540 nm, EM: 660 nm). The red channel was used to detect PI fluorescence.

The Nexcelom counting slide (CHT4-SD100) contains two chambers that each holds 20 μL. Each chamber has two small circular holes on the top, allowing injection of cells through one and air escape out the other. After filling the chambers with cell sample, the slide is inserted into the Cellometer system for image acquisition and analysis.

### Initial visual observation of TB- or PI-stained Jurkat cells

A sample of Jurkat cells (10 mL) was collected from cell culture and stored at 4°C for 2 days to allow the cells to naturally die. After 2 days, a 20-μL aliquot was retrieved and stained 1:1 with 0.4% TB or PI. Half of the stained cell sample was pipetted into a counting chamber and imaged using the Cellometer AutoT4 and AutoM10 in bright field. The captured images were qualitatively assessed for cell morphology and population to differentiate between TB- and PI-stained Jurkat cells. The initial visual observation was conducted three times.

### Fluorescent image acquisition of TB and PI dual-stained Jurkat cells

The Cellometer Vision 10X was used to capture bright field and fluorescent images of TB- and PI-stained Jurkat cells. First, a 20-μL aliquot of a cell sample stored at 4°C for 1 day was stained 1:1 with PI (20 μL) and then pipetted into a counting chamber. It was imaged in both bright field and fluorescence to determine the proper exposure time (3000 ms) for dead cells. A sample stained with 0.4% TB was also imaged to check for auto-fluorescence. Finally, a sample (10 μL) was first stained with PI (10 μL) and then immediately stained with 0.4% TB (10 μL). The dual-stained sample was imaged under bright field and fluorescence to confirm the presence of nuclei in the diffuse objects. Observation of dual-stained Jurkat cells with TB and PI was conducted two times.

### Video acquisition of TB and PI staining dead or dying cells

**Fig 1:**
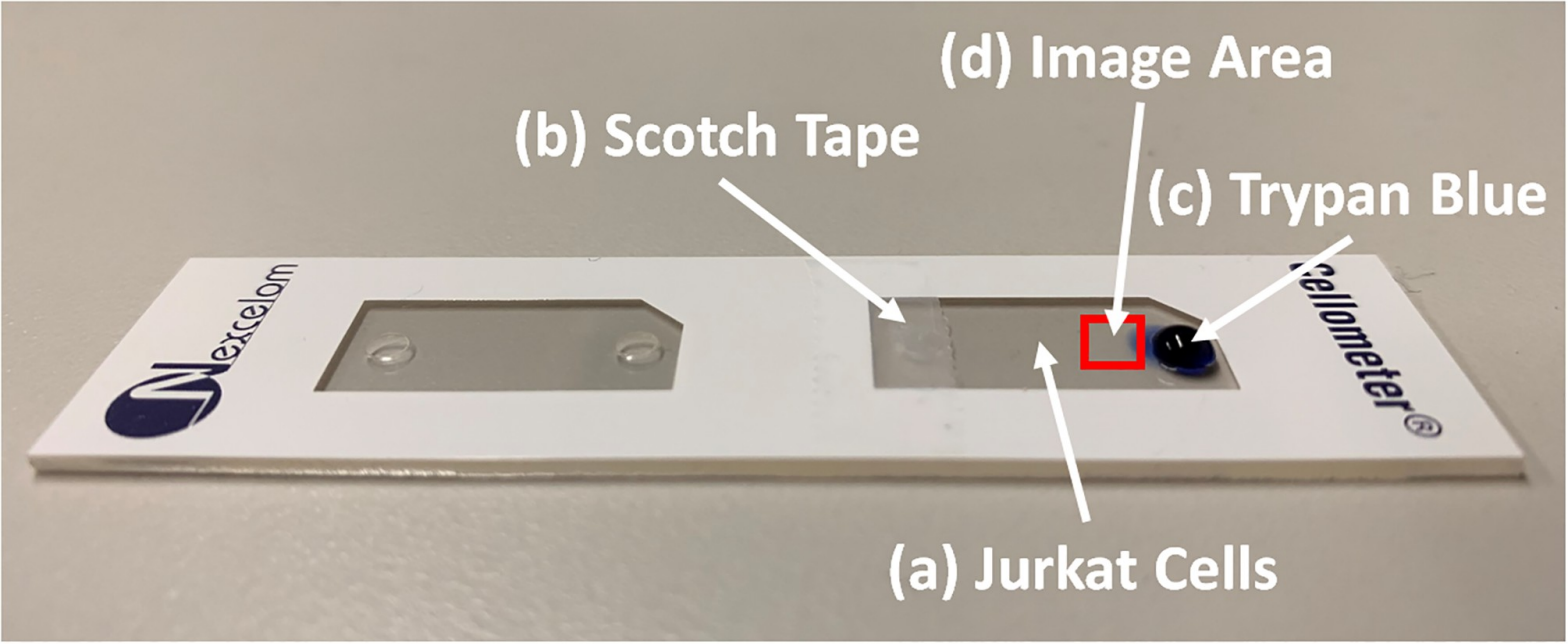
Counting chamber setup for video acquisition. (a) A Nexcelom counting chamber is first filled with 20 μL of Jurkat cells. (b) A small piece of Scotch tape is then placed over the air escape hole to inhibit flow of liquid in the chamber. (c) Next, a 10 μL aliquot of trypan blue is pipetted into the inlet hole to allow diffusion into the chamber. (d) The red square indicates the location were the interaction between Jurkat cells and trypan blue is recorded.

Both Cellometer AutoT4 and AutoM10 instruments were physically opened to allow access to the stage that holds the counting slide. Jurkat cells (stored at 4°C for 1 day) were pipetted into a counting chamber, and a small piece of Scotch tape was used to block one of the two holes on the chamber. Next, the counting chamber was placed directly under the objective lens for video acquisition. The Camtasia software suite (TechSmith, Okemos, MI) was set up to capture the imaging screen. Subsequently, ~10 μL of 0.4% TB was pipetted onto the top of the open hole in the filled counting chamber, which allowed slow diffusion of TB into the cell sample. The counting slide setup is shown in Fig 1. The slide was recorded for 1–10 min to show the effects of TB on dead Jurkat cells. The same process was repeated for PI. The videos were sped up with Adobe After Effect software (San Jose, CA). Video recording was conducted two times for TB and PI staining Jurkat cells.

### Time-course analysis of naturally-dying Jurkat cell populations

A naturally-dying Jurkat cell sample was produced by transferring 5 mL of cells (~4 x 10^6^ cells/mL) in one T25 flask, and placed into a bench-top drawer. The flask was kept in the drawer at room temperature for the remainder of the experiment. A small aliquot (200 μL) of cells was removed from the flask under sterile conditions at t = 0, 6, 12, 24, 48, 72, 96, and 168 h, stained 1:1 with 0.4% TB (Sigma-Aldrich, St. Louis, MO), and then analyzed using the AutoT4 at n = 4. The software was used to determine the concentration and population percentages of the dead/dying cell population at different range of sizes (5–8, 8–12, and 12–30 μm). The experiment was conducted three times.

### Time-course analysis of naturally-dying mouse bulk splenocyte populations

Fresh mouse bulk splenocyte sample was a kind donation from Christina A. Kuksin at the University of Massachusetts Amherst. Lysing protocol was first performed to lyse the red blood cells in the splenocyte sample. Cells were spun down and re-suspended in 1 mL of ACK lysing buffer (Life Technologies, Carlsbad, CA). After 5 min of incubation the cells were again spun down and re-suspended in RPMI. The splenocytes were stored in room temperature and collected at 0, 33, and 50 h. The cells were stained 1:1 with 0.1% TB and analyzed on the Vision 5X at n = 4. Similarly, the software was used to determine the concentration and population percentages of the dead/dying cell population at different range of sizes (2–5, 5–9, and 9–30 μm). The experiment was conducted one time due to the availability of mouse splenocytes.

### Time-course analysis of naturally-dying PBMC populations

Frozen PBMCs were purchased from Stemcell Technologies (Vancouver, Canada). The PBMCs were thawed and washed following manufacturer instructions. After washing, the PBMCs were resuspended in 4 mL of RPMI media in a T25 flask and incubated overnight at 37°C with 5% CO_2_. Approximately 1 mL of the PBMCs was removed and pipetted into a sterile T25 flask and stored at room temperature and collected at 0, 24, 48, and 72 h. The PBMCs were stained 1:1 with 0.2% TB and analyzed on the AutoT4 at n = 4. Similarly, the software was used to determine the concentration and population percentages of the dead/dying cell population at different range of sizes (3–9, 9–13, and 13–30 μm). The experiment was conducted one time.

In addition, the AutoM10 was used to record the formation of the diffuse objects described previously. The slide was recorded for 10 min to show the effects of TB on dead PBMCs. The video was sped up similarly.

### Effects of buffer concentration on the cell rupturing phenomenon with TB

To investigate the potential cause of cell rupturing, we resuspended the Jurkat cells in different concentrations of phosphate-buffered saline (PBS) to examine the effects of water influx. One-day-old Jurkat cells were collected and separated into seven 15-mL centrifuge tubes at 2 mL/tube. The samples were centrifuged and resuspended in 0.25, 1, 2, 4, 6, 8, or 10X concentrated PBS, as well as a control with media only. Next, each sample was stained 1:1 with 0.2% TB, and images were immediately captured using AutoM10. The difference of light intensity of TB-stained Jurkat cells and background (Δ*Intensity* = *Intensity*_*Background*_ − *Intensity*_*Cell*_) were measured using ImageJ from the captured images.

The experiment was repeated by resuspending Jurkat cells in different concentrations of sucrose. Two-day-old Jurkat cells were collected and separated into eight micro-centrifuge tubes at 400 μL/tube. The samples were centrifuged and resuspended in 9.4, 18.8, 37.5, 75.0, 150.0, 300.0, 1500.0, or 3000 μM of sucrose. Next, each sample was stained 1:1 with 0.2% TB, and images were immediately captured using AutoT4. The light intensity of TB-stained Jurkat cells were measured using ImageJ from the captured images. Both PBS and sucrose experiments were conducted two times.

### Video and image acquisition of TB effects on heat-killed Jurkat cells

Video and image acquisition on the AutoM10 were performed for a heat-killed sample to observe differences with naturally dying cells. A fresh Jurkat cell sample was aliquoted (10 mL) into a 50-mL tube and then placed into boiling water on a hot plate for 10 min. Next, 1 mL of the heat-killed cells were mixed with 1 mL of fresh Jurkat cells. The sample was pipetted into a counting chamber as described above. Subsequently, ~10 μL of 0.4% TB was pipetted on to the open hole, and the slide was recorded for 10 min. The final video was sped up similarly. Still images were acquired for the mixed Jurkat cells stained with TB for a final comparison. Cellometer Vision 10X was also used to capture the mixed Jurkat cells double-stained with PI and TB. Video recording was conducted two times for TB staining heat-killed Jurkat cells.

## Results

### Visual analysis of TB-stained Jurkat cell morphologies

**Fig 2:**
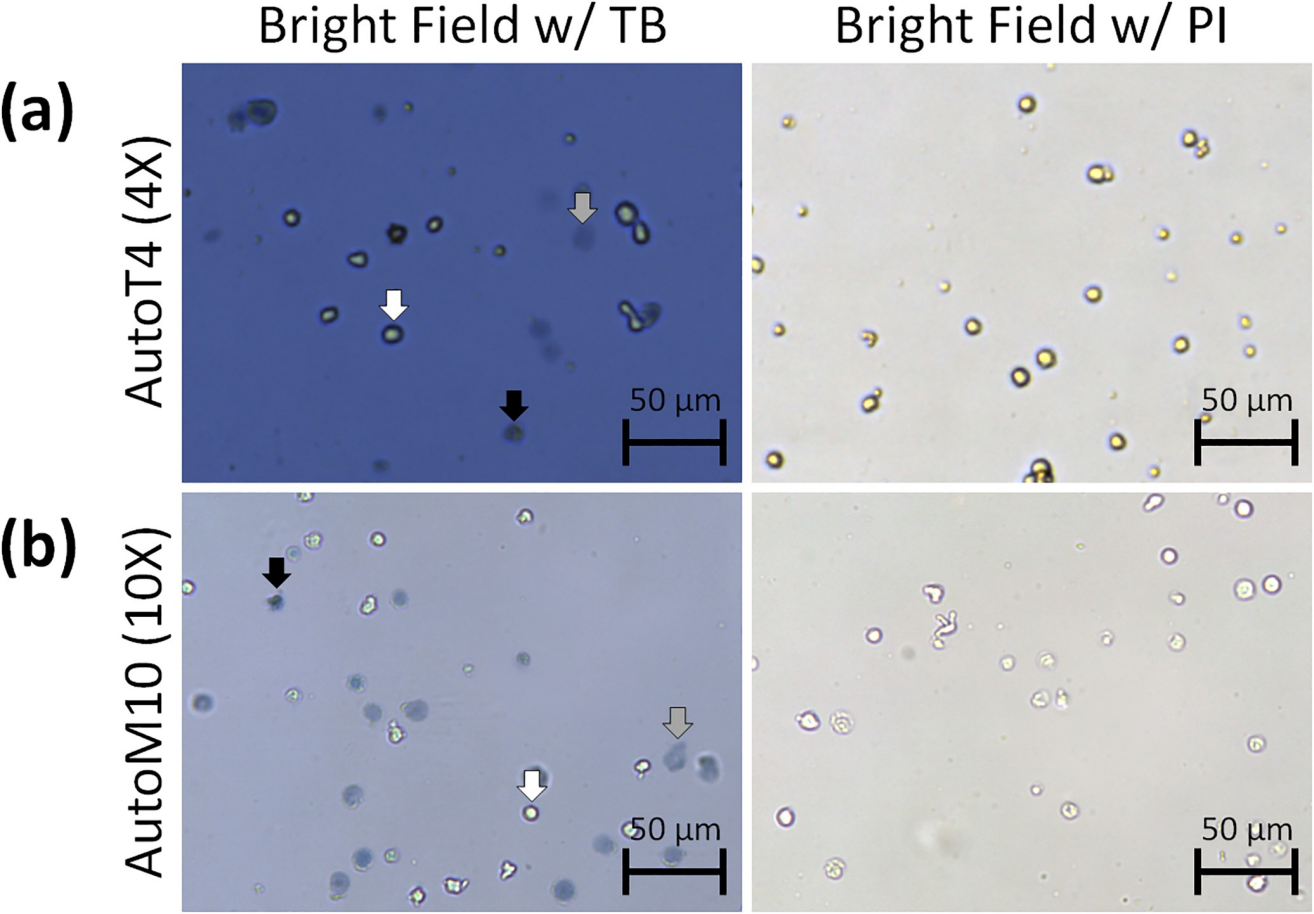
Bright field images of 2-day-old Jurkat cells stained with trypan blue or propidium iodide. The cells were imaged using the Cellometer AutoT4 (top) or AutoM10 (bottom). Trypan blue-stained Jurkat cells were classified into three groups: live (white arrow), dead (black arrow), and diffuse (gray arrow). The diffuse objects at 10X magnification showed bright materials surrounding them, resembling ruptured cell membrane. Cells stained with propidium iodide did not exhibit the same diffuse morphology as trypan blue-stained cells.

The bright field images of 2-day-old Jurkat cells captured with the AutoT4 and AutoM10 immediately after TB staining showed three distinct morphological populations (Fig 2). Cells that were bright, round, and plump were live cells not stained by TB. Cells that were blue, dark, and compact are dead and stained by TB. Large, dim, and diffuse objects were potentially dead or dying cells that were affected by TB, as previously described [13]. Jurkat cells stained with PI did not exhibit the same morphological changes. Round cells with thick membranes and bright centers were most likely alive, while smaller cells with low bright field contrast were potentially dead. The imaging results confirm the morphological effects of TB on cell samples and the lack of effects with PI. The AutoT4 at 4X magnification was initially utilized to capture images and videos of ruptured cells (Fig 2a). The AutoM10 at 10X magnification was used to enhance the resolution and identify more detailed features of the ruptured cells (Fig 2b). The 10X magnification showed some bright material surrounding the diffuse objects, which resembled the ruptured cell membrane. The bright field images acquired by AutoT4 and AutoM10 showed different background light intensity, which was dependent on the magnification of the system, and did not interfere with image analysis. It is important to note that the initial visual and quantification experiments utilized Jurkat cells (immortalized T lymphocytes) as a cell model for our experiments because these cells better mimic the cells used in cellular therapy.

### Visual proof that diffuse TB-positive objects are dead cells

**Fig 3:**
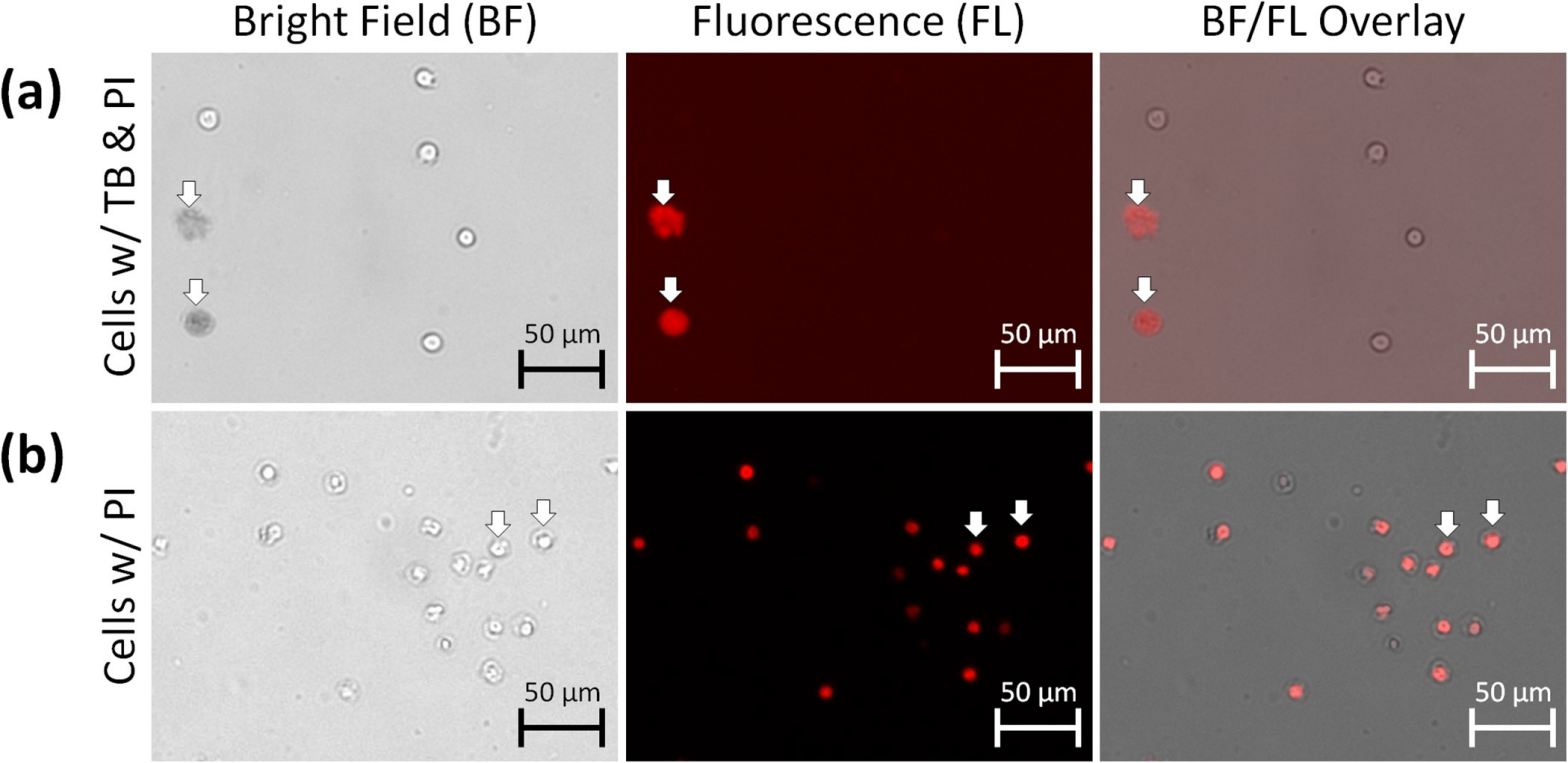
Bright field and fluorescent overlay images of 1-day-old Jurkat cells stained with propidium iodide with or without trypan blue. The cells were imaged using the Cellometer Vision 10X. (a) Co-localization of propidium iodide and trypan blue confirmed that dim and diffuse objects were dead cells. (b) Jurkat cells stained with propidium iodide retained cell membrane morphology.

To prove that dim and diffuse objects are dead cells, a test was conducted to determine if these objects contain nuclear DNA. First, we showed that PI-stained dead cells fluoresce brightly at the set exposure time of 3000 ms, and then showed that TB-stained objects did not auto-fluoresce in the red channel at the same exposure time, eliminating the uncertainty of fluorescent signals when cells are dual-stained with TB and PI (S2 Fig). Finally, dual TB and PI staining showed an overlap between the PI fluorescent signal and TB-positive objects, indicating that the diffuse TB-stained objects were in fact dead cells (Fig 3a). Merged bright field and red fluorescence images show that PI-positive cells retained a compact appearance and did not appear to exhibit the diffuse morphology of TB-positive cells (Fig 3b), which suggests that PI does not have the same morphological effects as TB.

### Video evidence of TB rupturing dead or dying Jurkat cells

After confirming that the TB-positive diffuse objects were all PI-positive, we recorded the morphological transformation of 1-day old Jurkat cells during TB staining. S1 Video (~30 s corresponding to 90 s in real time) showed TB diffusing across the screen from bottom to top, which changed the cells to large diffuse objects (from ~8 to 26 μm) within 45 s after interacting with TB. S2 Video (~6 s at normal speed) showed TB diffusing from top to bottom, which illustrated cell rupturing within ~2 s and disappearing toward the end. S3 Video (~30 s corresponding to 10 min in real time) showed TB-induced morphological changes in dead Jurkat cells at ~7 min. Visually, some dead cells were rendered dark and compact, while others were dim and diffuse. Finally, PI-staining of Jurkat cells is shown in S4 Video (~30 s corresponding to 10 min in real-time), resulting in no observable morphological changes.

### Formation of diffuse TB-stained Jurkat cell population over time

**Fig 4:**
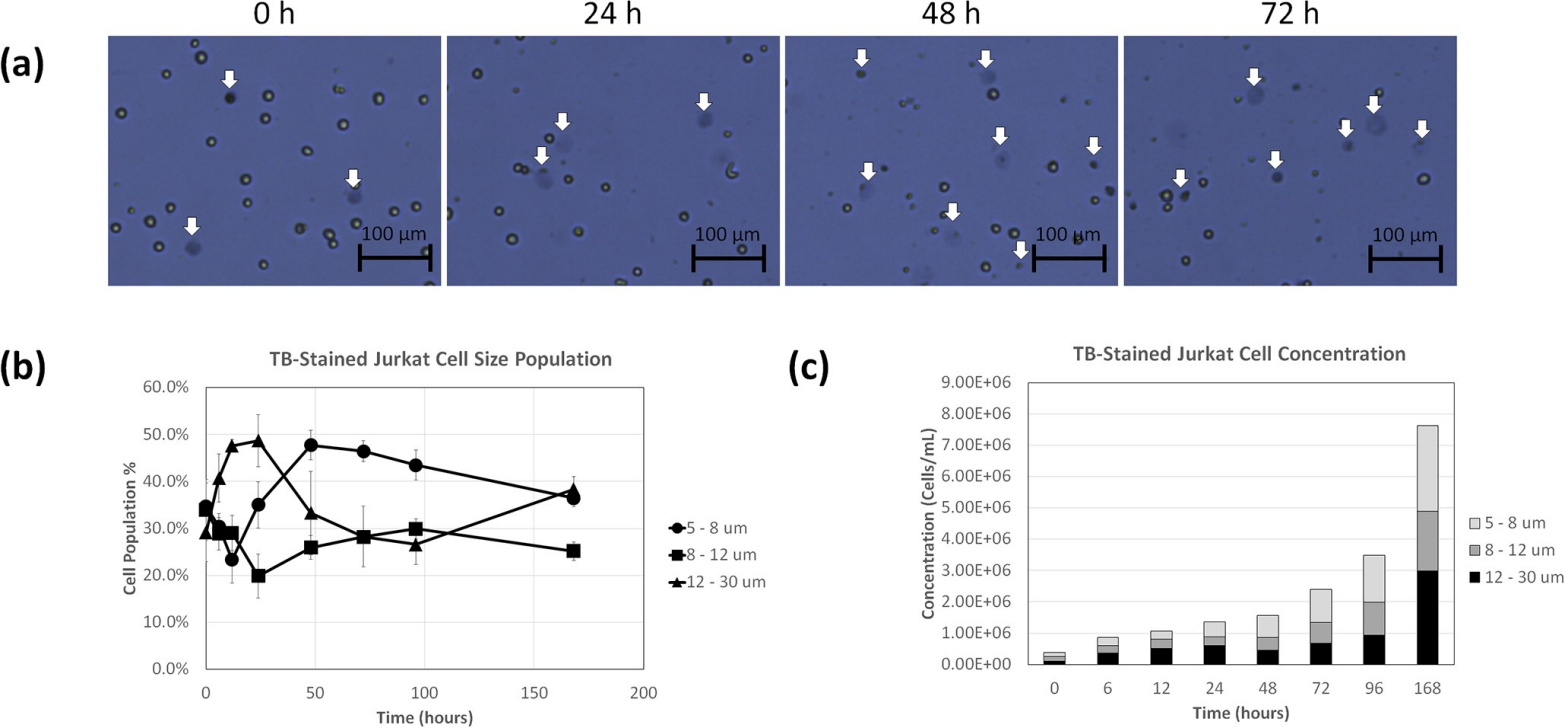
Time-course morphological analysis of TB-stained Jurkat cells. (a) Bright field images of TB-stained Jurkat cells acquired by the AutoT4 showing diffuse objects. (b) Time-dependent size populations over 168 h, showing increase in diffuse objects. (c) Concentration changes for different size populations over time.

The morphological changes of TB-stained Jurkat cells were monitored over 168 hours to quantify the change in the diffuse dead/dying cell concentration and population percentages. The time-dependent bright field images of TB-stained Jurkat cells are shown in Fig 4a, which displayed the change in the number of diffuse cells over time. The average cell size results with standard deviation (Fig 4b) of TB-stained Jurkat cells showed increase in the diffuse population (12–30 μm) in the first 24 hours, and gradually decreased over time (n = 4 cell samples). On the other hand, the cells typically counted (8–12 μm) showed slight increase over time. Finally, a small size population (5–8 μm) showed significant increase over time that represented cell fragments and debris. Correspondingly, the TB-stained Jurkat cell concentrations increased over the 168 hours (Fig 4c). Approximately 100–500 cells were counted to generate the cell size graphs.

### Formation of diffuse TB-stained mouse splenocyte population over time

**Fig 5:**
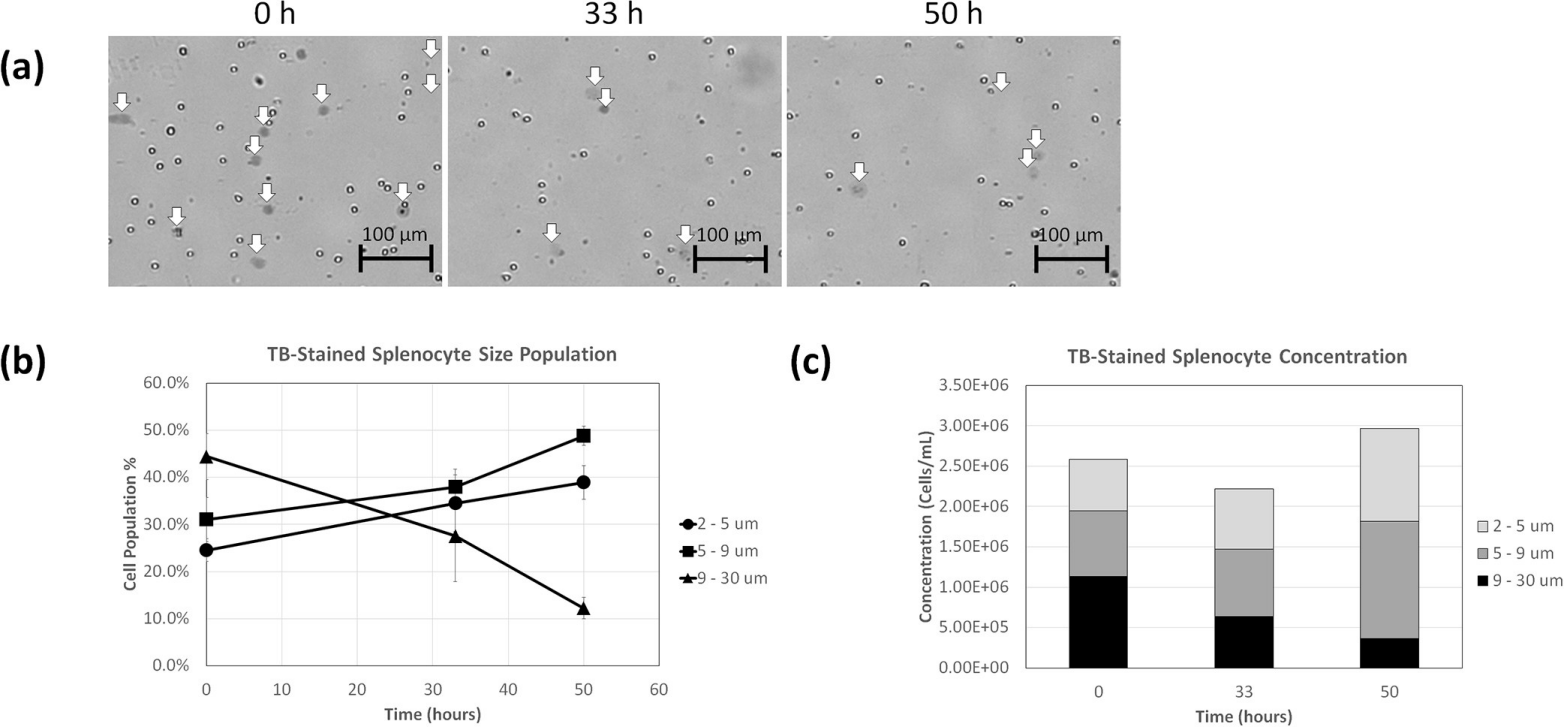
Time-course morphological analysis of TB-stained mouse splenocytes. (a) Bright field images of TB-stained mouse splenocytes acquired by the Vision 5X showing diffuse objects. (b) Time-dependent size populations over 50 h, showing increase in diffuse objects. (c) Concentration changes for different size populations over time.

The morphological changes of TB-stained mouse splenocytes were monitored over 50 hours to quantify the change in the diffuse dead/dying cell concentration and population percentages. The time-dependent bright field images of TB-stained mouse splenocytes are shown in Fig 5a, which displayed the change in the number of diffuse cells over time. The average cell size results with standard deviation (Fig 5b) of TB-stained mouse splenocytes showed decrease in the diffuse population (9–30 μm) over 50 hours (n = 4 cell samples). On the other hand, the cells typically counted (5–9 μm) and fragmented cells (2–5 μm) showed increase over time. Correspondingly, the TB-stained mouse splenocyte concentrations increased slightly over the 50 hours (Fig 5c). Approximately 100–500 cells were counted to generate the cell size graphs.

### Formation of diffuse TB-stained PBMC population over time

**Fig 6:**
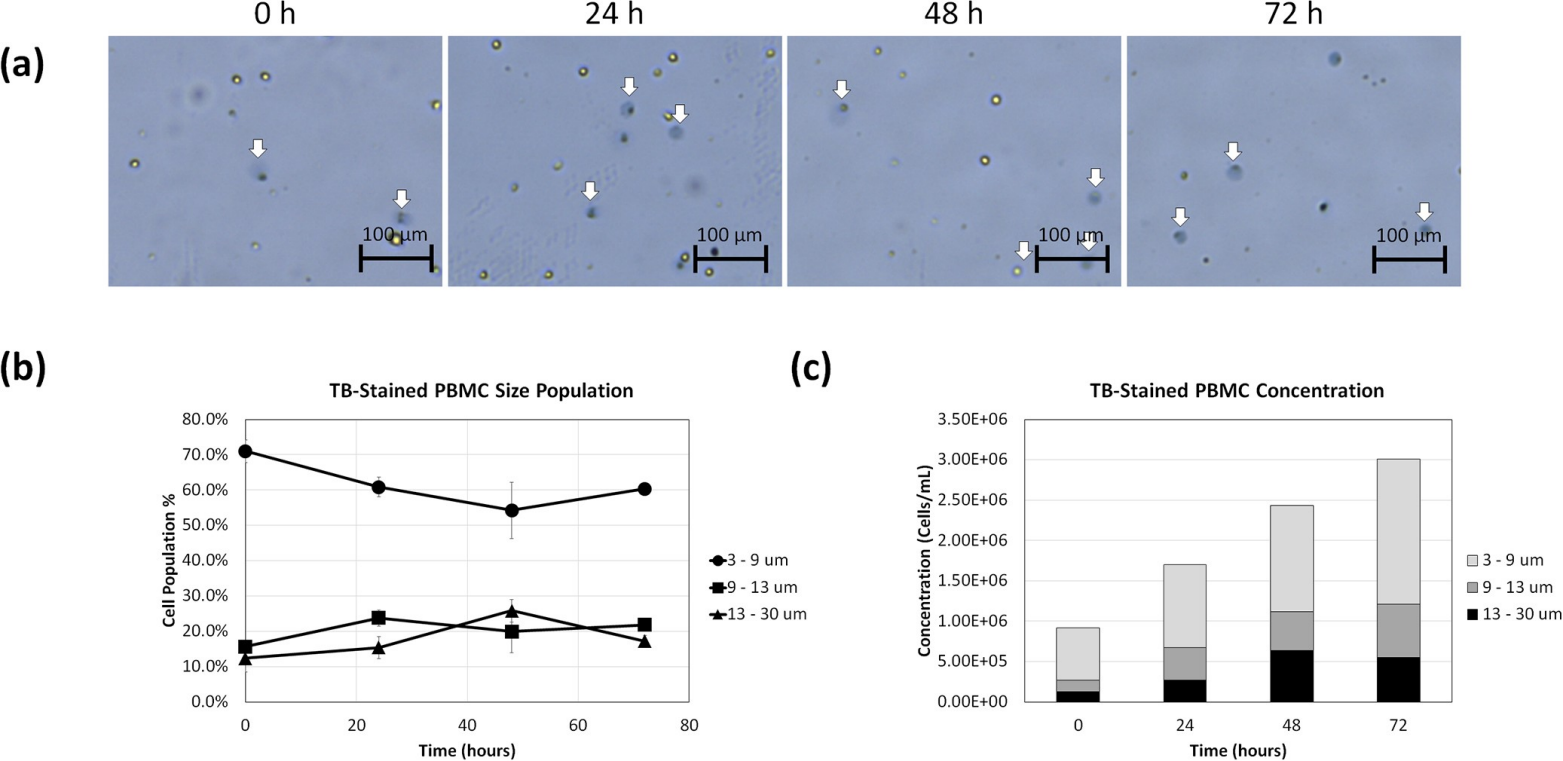
Time-course morphological analysis of TB-stained PBMCs. (a) Bright field images of TB-stained PBMCs acquired by the AutoT4 showing diffuse objects. (b) Time-dependent size populations over 72 h, showing increase in diffuse objects. (c) Concentration changes for different size populations over time.

The morphological changes of TB-stained PBMCs were monitored over 72 hours to quantify the change in the diffuse dead/dying cell concentration and population percentages. The time-dependent bright field images of TB-stained PBMCs are shown in Fig 6a, which displayed the change in the number of diffuse cells over time. The average cell size results with standard deviation (Fig 6b) of TB-stained PBMCs showed slight decrease in the normal and diffuse populations (9–30 μm) over 72 hours (n = 4 cell samples). On the other hand, the fragmented cells (3–9 μm) showed high starting percentages and decreased over time. Correspondingly, the TB-stained PBMC concentrations increased significantly over the 72 hours (Fig 6c). S5 Video showed the morphological changes to the PBMC during TB staining. Approximately 100–200 cells were counted to generate the cell size graphs.

### TB-based cell rupturing is caused by water influx

**Fig 7:**
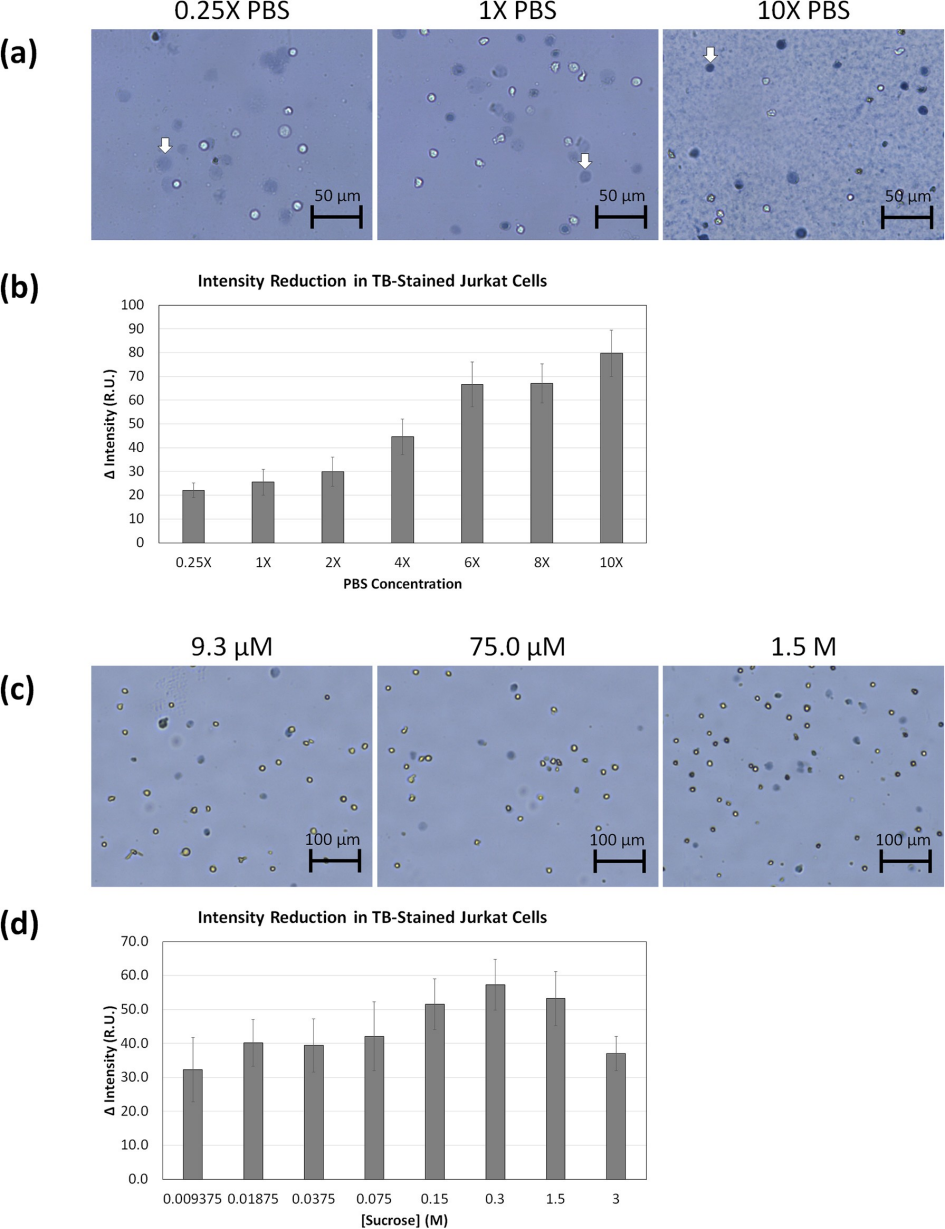
Bright field images and trypan blue intensity analysis of aged Jurkat cells resuspended in different buffer concentrations. (a) Jurkat cells were resuspended in 0.25, 1, 2, 4, 6, 8, or 10X concentrated PBS, and imaged using the AutoM10. The white arrows indicated the change from diffuse to dark objects as PBS concentration increased, (b) which was also validated with ImageJ intensity measurement of the TB-stained cells. A significant precipitation formed when exposed to high salt concentrations with trypan blue. (c) Jurkat cells were resuspended in 9.4, 18.8, 37.5, 75.0, 150.0, 300.0, 1500.0, or 3000 μM of sucrose, and imaged using the AutoT4. Similarly, the bright field images showed increase in TB darkness as sucrose concentration increased, (d) which again was validated by the ImageJ intensity analysis.

We hypothesized that cell rupturing may be due to higher osmotic pressure causing rapid influx of water. Different PBS concentrations had obvious effects on TB staining (Fig 7a). The use of 0.25X PBS led to increases in the number and size of ruptured cells. Similar to cells in medium, those incubated in 1X to 6X PBS had similar diffuse morphology but with increasing darkness. The 8X and 10X PBS formed precipitates, presumably due to high salt concentration [17], but reduced the number and size of ruptured cells, with a significant increase in darkness. The PBS concentration-dependent darkness is shown in Fig 7b, which showed the reduction in light intensity as the PBS concentration increased. These results strongly indicate that cells ruptured due to water influx during TB staining.

Similarly, when Jurkat cells were resuspended in various concentrations of sucrose, we observed the reduction in light intensity as the sucrose concentration increased (Fig 7c and 7d), indicating increasing TB molecules in the dead/dying cells. However, the light intensity reduction decreased at the two highest sucrose concentrations, which could be due to the density of solution preventing proper mixing with TB.

### Heat-killed dead cells present different TB staining behaviors

**Fig 8:**
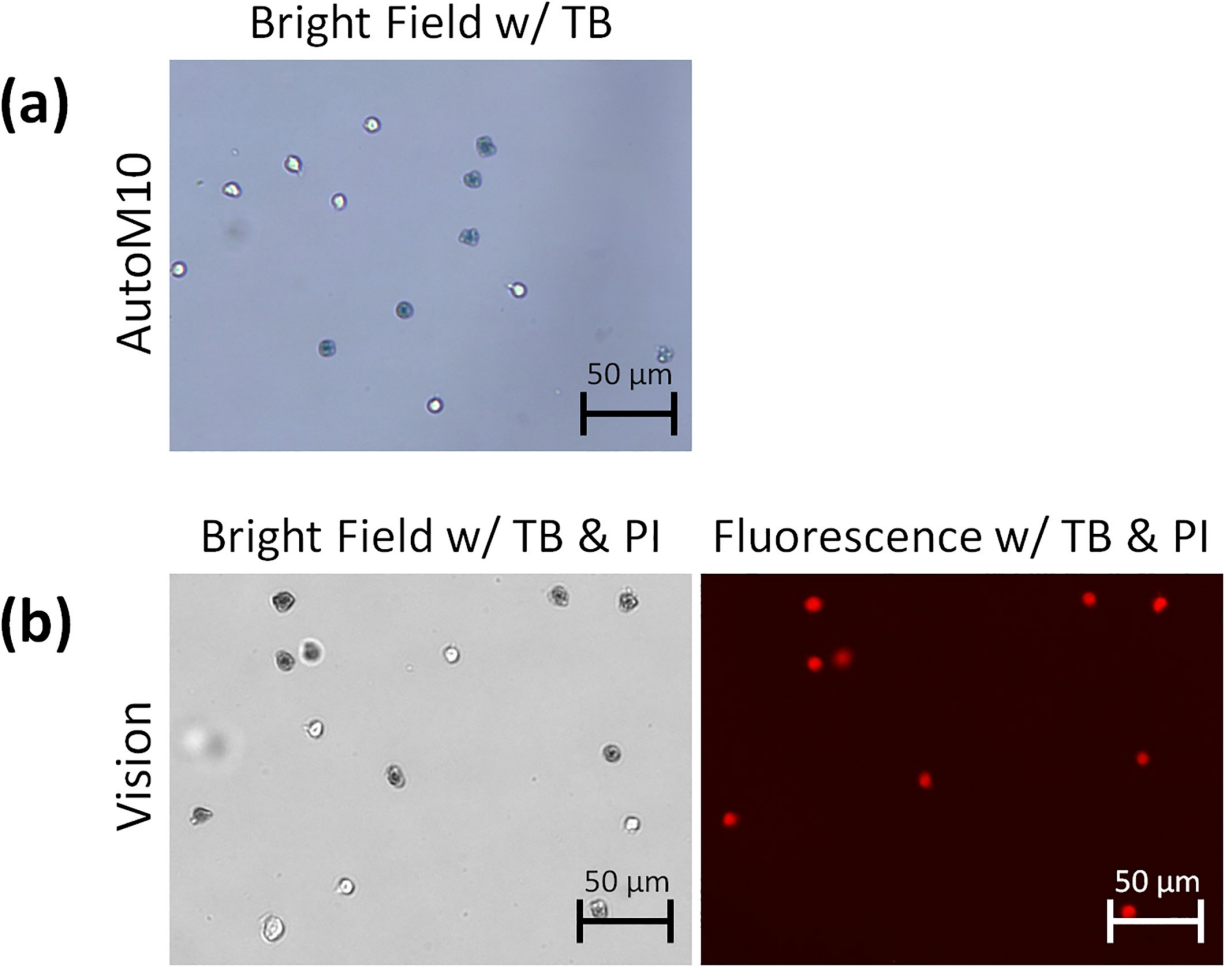
Bright field and fluorescent images of a 1:1 mixture of fresh and heat-killed Jurkat cells stained with trypan blue with or without propidium iodide. The cells were imaged using both AutoM10 and Vision 10X. (a) Unlike naturally dying cells, no membrane rupturing was observed, and heat-killed Jurkat cell morphology remained tight and dark when stained with trypan blue. (b) Co-localization of trypan blue and propidium iodide in heat-killed Jurkat cells.

We previously showed that heat-killed Jurkat cells stained with TB did not exhibit diffused morphology [13,18]. Similarly, these cells showed a very different staining pattern than naturally dying cells. In S6 Video, TB appeared to enter the dead cells and increase the darkness without rupturing the membranes (10X, ~30 s corresponding to 10 min in real time). Cell size analysis revealed that the change was less than 1 μm. The final still images showed no expansion of TB-stained cells (Fig 8a), and PI fluorescence was emitted by dual-stained samples (Fig 8b). It is important to note that we utilized the mixture of live and heat-killed Jurkat cells to demonstrate morphological distinction compared to naturally dying cells when stained with trypan blue.

## Discussion

Cellular therapies have become important cancer treatments due to their efficacy and the 2017 approval of two chimeric antigen receptor (CAR) T cell therapies by the FDA [19]. CAR T cell therapy requires gene editing of T cells from patients, expanding the culture, and infusing the final products back into the patients. Accurate cell counting is needed to ensure proper dosages are administered. More critically, cell products must have acceptable viability to minimize the risk of an autoimmune response [1,2], where the FDA recommends greater than 70% viability for cellular therapy products [20]. We hypothesize that transferring a large amount of nonviable cells to the patients may pose some potential serious side effects such as cytokine release syndrome [21].

Since 2012, we have scientifically identified and published two articles related to cell counting issues with TB staining. The first revealed that bright field counting with TB can over-count live peripheral blood mononuclear cells [22]. The second publication quantitatively showed that TB can cause morphological changes in dead or dying cells, transforming them to dim and diffuse shapes for Jurkat cells and primary mouse splenocytes [13]. The origin and identity of these objects have been since unanswered. In this work, we present visual evidence of their formation, as well as a potential explanation of why they form in the presence of TB.

Biologists typically stain cells 1:1 with TB at concentrations ranging from 0.05% to 1% [23], which may cause inconsistencies in the identification of dead or dying cells as we previously reported [13]. Stained cells are observed under bright field when the diffused objects have already formed and/or disappeared, thus they are not included in the dead cell count. Traditionally, diffuse objects were difficult to see in microscopy images, causing researchers to under-count dead cells and over-estimate viability. In contrast, the optical components in Cellometer instruments were able to clearly image the dim and diffuse objects at various trypan blue concentrations and instruments.

We were able to set up the cell counting chamber and allow TB to diffuse in, so the interaction between cells and dye could be observed and recorded. The videos clearly showed that some cells stained with TB immediately ruptured and formed the diffuse objects (S1 and S2 Videos), while PI (S4 Video) did not induce the same morphological changes (S3 Fig). In Video 1–3, cells ruptured over different time frames; some ruptured immediately after contacting TB, some were affected in less than 20 s, and some ruptured more than 5 min later. This could be due to the TB diffusion rate in the counting chamber. PI staining showed that the diffuse objects contained DNA, confirming they were cells. The live cell appearance was not affected by TB or PI, thus both methods yielded comparable live cell counts as previously reported [13].

The diffuse objects were quantified over time to demonstrate that as cells become unhealthy, they become more fragile which allowed the TB to induce the formation of more diffuse cells. The Jurkat cells were initially at high viability, and in the first 24 hours, the diffuse objects increased from 30–50%, which corresponded to the images. However, although the diffuse TB-stained cell concentration increased, the percentages did not continue to increase, which could be due to the lack of whole intact cells in the samples readily affected by TB. On the other hand, the initial condition of the primary mouse splenocytes was already showing large amount of diffuse objects potentially due to the preparation procedure and viability. Finally, primary human PBMCs initially showed large amount of fragmented cells and debris, which may require more washing steps. We were able to observe the formation of large diffuse objects (S5 Video) over time. It is important to note that Jurkat cells, primary mouse splenocytes, and PBMCs were selected as the representation of immune cells typically used in the discovery phase of cell therapy.

Cell rupturing can be mainly attributed to rapid water influx of water. We investigated whether osmotic pressure played a critical role in cell rupturing in the presence of TB by resuspending cells in hypertonic, isotonic, and hypotonic buffers. We hypothesized that binding with TB rapidly increased the number negatively charged residues on cytoplasmic proteins [16,24], which attracted more positively charged ions such as sodium, and this led to high water influx due to osmotic pressure that ruptured the already fragile cell membrane and cytoskeleton [3,25–27]. We observed increase in darkness in dead/dying Jurkat cells as PBS concentration increased, which indicated improvement in TB staining caused by high salt content in the buffer. In addition, the experiment was performed with various concentrations of sucrose, which is another method of changing the osmotic pressure of the buffer solution. The results again showed increased darkness in TB-stained Jurkat cells as sucrose concentration increased. However, the effects were not as dramatic as with a salt solution, which indicated that the rupturing may be affected more by the ionic strength of the media or buffer. Interestingly, the heat-killed cells did not rupture by TB (S6 Video), which may be caused by the denaturing of membrane proteins during boiling, resulting in cell membrane hardening that can withstand the influx of TB and water [28,29]. Therefore, in order to generate cell conditions that can form diffuse objects after TB staining, we allowed the cells to die in an unfavorable environment. We observed that the diffuse objects typically form at lower cell viabilities for immune cells. In contrast, we did not observe the similar morphological changes for typical cancer cell cultures at high viabilities.

It is important for biologists to select the most accurate cell counting method that is fit-for-purpose, especially for cellular therapy products such as CAR T, where cell number and viability significantly affect treatment efficacy. Cells that rupture during TB staining are difficult to see under bright field imaging and can be under-counted, leading to an over-estimation of cell viability. We suggest using fluorescent nuclear staining methods that may estimate cell viability more accurately [13,18,22,30–32]. Future work will involve in developing a method to validate cell viability measurement, as well as identifying different cell populations relating to the dying process [33] to further improve the characterization of cell fitness and death.

## Supporting information

S1 Video Bright field video captured with AutoT4 (4X, ~30-s time lapse corresponding to 90 s in real time) of staining Jurkat cells with trypan blue. The Jurkat cell in the red circle immediately became diffuse after interacting with trypan blue. Numerous dead Jurkat cells were stained with trypan blue and transformed into diffuse objects.
(MP4)

S2 Video Bright field video captured with AutoM10 (10X, ~8 s in real time) of staining Jurkat cells with trypan blue. Resolution was improved at 10X magnification. The Jurkat cells in the red circle immediately ruptured after contact with trypan blue. Many other cells clearly developed large and diffuse morphology.
(MP4)

S3 Video Bright field video captured with AutoM10 (10X, ~30-s time lapse corresponding to 10 min in real time) of staining Jurkat cells with trypan blue. The Jurkat cells in the red circle again showed staining and morphological transformation after contact with trypan blue. The cells clearly expanded into large and diffuse shape.
(MP4)

S4 Video Bright field video captured with AutoM10 (10X, ~30-s time lapse corresponding to 10 min in real time) of Jurkat cells staining with propidium iodide. Unlike trypan blue, the video showed no morphological changes to cells staining with propidium iodide within 10 min shown in the red circle.
(MP4)

S5 Video Bright field video captured with AutoM10 (10X, ~25-s time lapse corresponding to 2 min in real time) of staining PBMCs with trypan blue. The PBMCs in the red circle immediately ruptured after contact with trypan blue.
(MP4)

S6 Video Bright field video captured with AutoM10 (10X, ~30-s time lapse corresponding to 10 min in real time) of staining heat-killed Jurkat cells with trypan blue. The red circle in the video indicates dead heat-killed Jurkat cells stained with trypan blue without rupturing the cell membrane.
(MP4)

S1 Fig Digitally captured bright field image from a light microscope using the 10X objective. The zoomed image shows three populations: bright, round, and plump (white arrow, live cell); blue, dark, and tight (black arrow, dead cell); and large, dim, and diffuse (gray arrow, ruptured dead cell).
(TIF)

S2 Fig Bright field and fluorescent images of 1-day-old Jurkat cells stained with trypan blue or propidium iodide and imaged using the Vision 10X. (a) Dead Jurkat cells stained with propidium iodide exhibited bright red fluorescence. (b) Dead Jurkat cells stained with trypan blue showed no background signal.
(TIF)

S3 Fig Time-course bright field images cropped from the videos. The progression of the images show the morphological changes to the cells staining with TB or PI.
(TIF)

S1 Data Measurement and analysis of morphological changes for TB-stained Jurkat cells. (XLSX)

S2 Data Measurement and analysis of morphological changes for TB-stained mouse splenocytes. (XLSX)

S3 Data Measurement and analysis of morphological changes for TB-stained human PBMCs. (XLSX)

S4 Data Measurement and analysis of the light intensity of TB-stained Jurkat cells in various concentrations of PBS. (XLSX)

S5 Data Measurement and analysis of the light intensity of TB-stained Jurkat cells in various concentrations of sucrose. (XLSX)

## References

[1] Nagata S, Hanayama R, Kawane K (2010) Autoimmunity and the Clearance of Dead Cells. Cell 140(5): 619–630. 10.1016/j.cell.2010.02.014 20211132

[2] Rock KL, Kono H (2008) The inflammatory response to cell death. Annual Review of Pathology 3: 99–126. 10.1146/annurev.pathmechdis.3.121806.151456 18039143 PMC3094097

[3] Zhang Y, Chen X, Gueydan C, Han J (2018) Plasma membrane changes during programmed cell deaths. Cell Research 28: 9–21. 10.1038/cr.2017.133 29076500 PMC5752838

[4] Arcidiacono JA, Bauer SR, Kaplan DS, Allocca CM, Sarkar S, Lin-Gibson S (2018) FDA and NIST collaboration on standards development activities supporting innovation and translation of regenerative medicine products. Cytotherapy 20: 779–784. 10.1016/j.jcyt.2018.03.039 29784433

[5] Page SW (2008) Chapter 10—Antiparasitic drugs In: Maddison J, Page S, Church D, editors. Small Animal Clinical Pharmacology (Second Edition): Saunders Ltd pp. 198–260.

[6] Piccinini F, Tesei A, Arienti C, Bevilacqua A (2017) Cell Counting and Viability Assessment of 2D and 3D Cell Cultures: Expected Reliability of the Trypan Blue assay. Biological Procedures Online 19(8): 1–12. 28814944 10.1186/s12575-017-0056-3 PMC5518102

[7] Beaudoin AR, Kahkonen D (1963) The Effect of Trypan Blue on the Serum Proteins of the Fetal Rat. The Anatomical Record 147(3): 387–395. 14077651 10.1002/ar.1091470310

[8] Black L, Berenbaum MC (1964) Factors Affecting the Dye Exclusion Test for Cell Viability. Experimental Cell Research 35: 9–13. 10.1016/0014-4827(64)90066-7 14190667

[9] Gao H-W, Zhao J-F (2003) Interaction of Trypan Blue with Protein and Application. Journal of the Chinese Chemical Society 50: 329–334.

[10] Kwok AKH, Yeung C-K, Lai TYY, Chan K-P, Pang CP (2004) Effects of trypan blue on cell viability and gene expression in human retinal pigment epithelial cells. British Journal of Ophthalmology 88(12): 1590–1594. 10.1136/bjo.2004.044537 15548818 PMC1772415

[11] Mascotti K, McCullough J, Burger SR (2000) HPC viability measurement: trypan blue versus acridine orange and propidium iodide. Transfusion 40(6): 693–696. 10.1046/j.1537-2995.2000.40060693.x 10864990

[12] Tennant JR (1964) Evaluation of the Trypan Blue Technique for Determination of Cell Viability. Transplantation 2(6): 686–694. 10.1097/00007890-196411000-00001 14224649

[13] Chan LL-Y, Kuksin D, Laverty DJ, Saldi S, Qiu J (2014) Morphological observation and analysis using automated image cytometry for the comparison of trypan blue and fluorescence-based viability detection method. Cytotech. 10.1007/s10616-014-9704-5 24643390 PMC4371569

[14] Tran S-L, Puhar A, Ngo-Camus M, Ramarao N (2011) Trypan Blue Dye Enters Viable Cells Incubated with the Pore-Forming Toxin HlyII of Bacillus cereus. PLOS One 6(9): e22876 10.1371/journal.pone.0022876 21909398 PMC3167804

[15] Chung DM, Kim JH, Kim JK (2015) Evaluation of MTT and Trypan Blue assays for radiation-induced cell viability test in HepG2 cells. International Journal of Radiation Research 13(4): 1–6.

[16] Wainwright M (2010) Dyes, trypanosomiasis and DNA: a historical and critical review. Biotechnic and Histochemistry 85(6): 341–354. 10.3109/10520290903297528 21080764

[17] Sarma KD, Ray D, Antony A (2000) Improved sensitivity of trypan blue dye exclusion assay with Ni^2+^ or Co^2+^ salts. Cytotechnology 32: 93–95. 10.1023/A:1008144527206 19002971 PMC3449693

[18] Chan LL, Wilkinson AR, Paradis BD, Lai N (2012) Rapid Image-based Cytometry for Comparison of Fluorescent Viability Staining Methods. Journal of Fluorescence 22(5): 1301–1311. 10.1007/s10895-012-1072-y 22718197

[19] Yip A, Webster RM (2018) The market for chimeric antigen receptor T cell therapies. Nature Reviews Drug Discovery 17: 161–162. 10.1038/nrd.2017.266 29375140

[20] Department-of-Health-and-Human-Services U.S., Food-and-Drug-Administration, Center-for-Biologics-Evaluation-and-Research (2008) Guidance for FDA Reviewers and Sponsors—Content and Review of Chemistry, Manufacturing, and Control (CMC) Information for Human Somatic Cell Therapy Investigational New Drug Applications (INDs)

[21] Hasegawa K, Hosen N (2019) Chimeric antigen receptor T cell therapy for multiple myeloma. Inflammation and Regeneration 39(10): 1–5. 31171941 10.1186/s41232-019-0100-6 PMC6547554

[22] Chan LLY, Laverty DJ, Smith T, Nejad P, Hei H, Gandhi R et al (2013) Accurate measurement of peripheral blood mononuclear cell concentration using image cytometry to eliminate RBC-induced counting error. Journal of Immunological Methods 388(1–2): 25–32. 10.1016/j.jim.2012.11.010 23201386

[23] Kofanova OA, Davis K, Glazer B, Souza YD, Kessler J, Betsou F (2014) Viable Mononuclear Cell Stability Study for Implementation in a Proficiency Testing Program: Impact of Shipment Conditions. Biopreservation and Biobanking 12(3): 206–216. 10.1089/bio.2013.0090 24955735 PMC4955601

[24] Cannon J, Kim D, Maruyama S, Shiomi J (2012) Influence of Ion Size and Charge on Osmosis. Journal of Physical Chemistry B 116(14): 4206–4211. 10.1021/jp2113363 22397596

[25] Bursch W, Hochegger K, Török L, Marian B, Ellinger A, Hermann RS (2000) Autophagic and apoptotic types of programmed cell death exhibit different fates of cytoskeletal filaments. Journal of Cell Science 113: 1189–1198. 10704370 10.1242/jcs.113.7.1189

[26] Ed Costa, Rodrigues EB, Dib E, Penha FM, Furlani BA, Magalhães O. J et al (2009) Vital Dyes and Light Sources for Chromovitrectomy: Comparative Assessment of Osmolarity, pH, and Spectrophotometry. Investigate Ophthalmology and Visual Science 50(1): 385–391. 10.1167/iovs.08-2285 18689696

[27] Lodish H, Berk A, Zipursky SL (2000) Section 15.8 Osmosis, Water Channels, and the Regulation of Cell Volume Molecular Cell Biology 4th edition New York: W. H. Freeman.

[28] Bischof JC, Padanilam J, Holmes WH, Ezzell RM, Lee RC, Tompkins RG et al (1995) Dynamics of Cell Membrane Permeability Changes at Supraphysiological Temperatures. Biophysical Journal 68: 2608–2614. 10.1016/S0006-3495(95)80445-5 7647264 PMC1282171

[29] Mackey BM, Miles CA, Parsons SE, Seymour DA (1991) Thermal denaturation of whole cells and cell components of Escherichia coli examined by differential scanning calorimetry Journal of General Microbiology 137: 2361–2374. 10.1099/00221287-137-10-2361 1722814

[30] Altman SA, Randers L, Rao G (1993) Comparison of Trypan Blue Dye Exclusion and Fluorometric Assays for Mammalian Cell Viability Determinations. Biotechnology Progress 9: 671–674. 10.1021/bp00024a017 7764357

[31] Solomon M, Wofford J, Johnson C, Regan D, Creer MH (2010) Factors influencing cord blood viability assessment before cryopreservation. Transfusion 50(4): 820–830. 10.1111/j.1537-2995.2009.02491.x 19919556

[32] Chan LL-Y, McCulley KJ, Kessel SL (2017) Assessment of Cell Viability with Single-, Dual-, and Multi-Staining Methods Using Image Cytometry. Methods in Molecular Biology. pp. 27–41. 10.1007/978-1-4939-6960-9_3 28470515

[33] Galluzzi L, Baehrecke EH, Chan FK-M (2018) Molecular mechanisms of cell death: recommendations of the Nomenclature Committee on Cell Death 2018. Cell Death and Differentiation 25: 486–541. 10.1038/s41418-017-0012-4 29362479 PMC5864239

