# Supplementary figures and images for: Observation and quantification of the morphological effect of trypan blue rupturing dead or dying cells
Source: PLoS One. 2020 Jan 24;15(1):e0227950. doi: 10.1371/journal.pone.0227950 (PMC6980413; doi:10.1371/journal.pone.0227950)

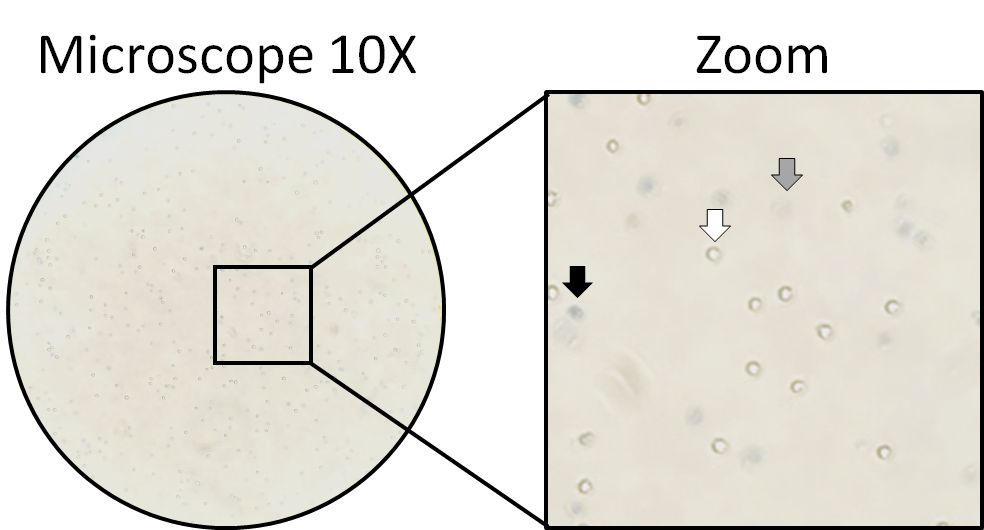

Supplement: S1 Fig — The zoomed image shows three populations: bright, round, and plump (white arrow, live cell); blue, dark, and tight (black arrow, dead cell); and large, dim, and diffuse (gray arrow, ruptured dead cell). (TIF) [file pone.0227950.s007.tif]

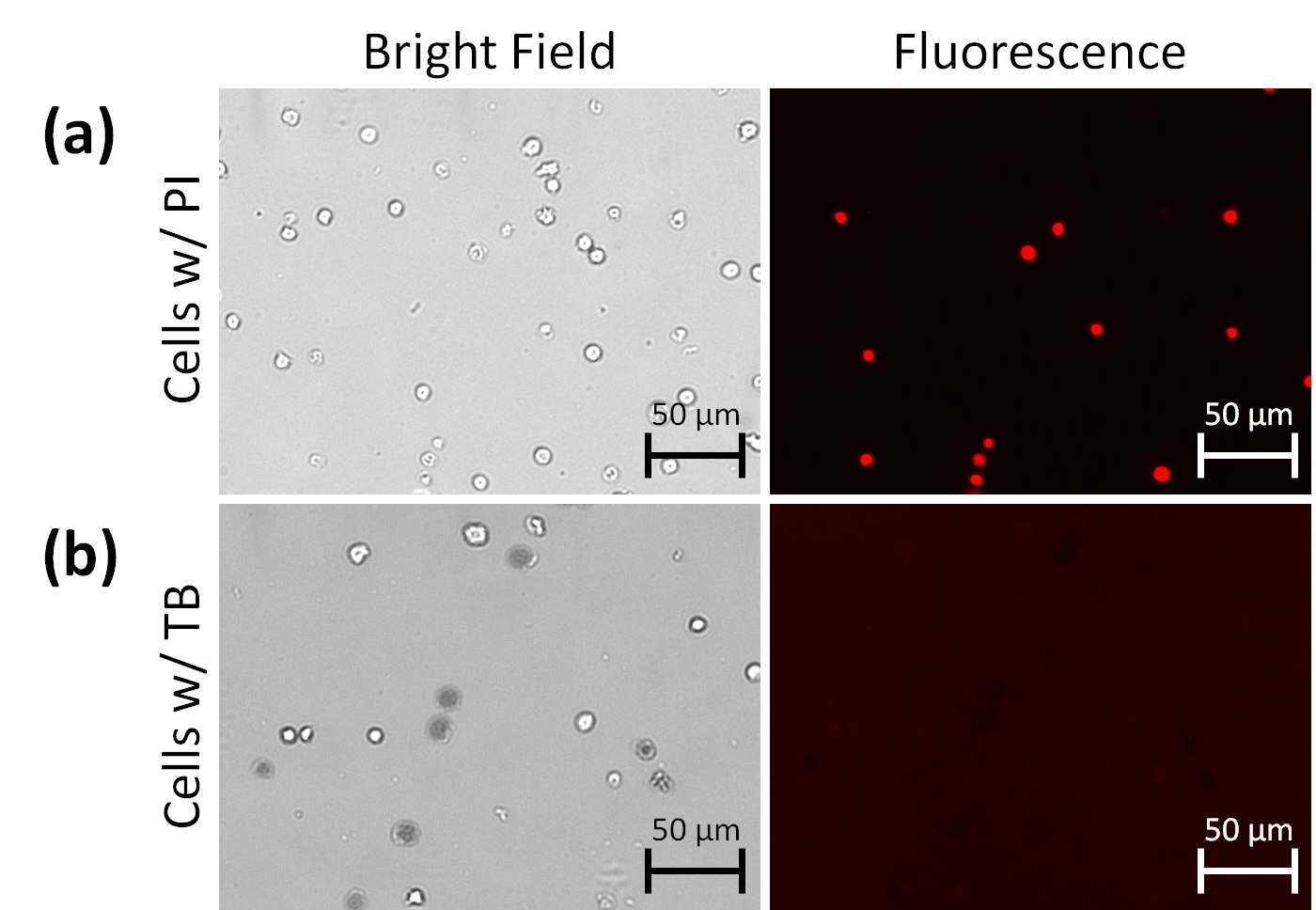

Supplement: S2 Fig — (a) Dead Jurkat cells stained with propidium iodide exhibited bright red fluorescence. (b) Dead Jurkat cells stained with trypan blue showed no background signal. (TIF) [file pone.0227950.s008.tif]

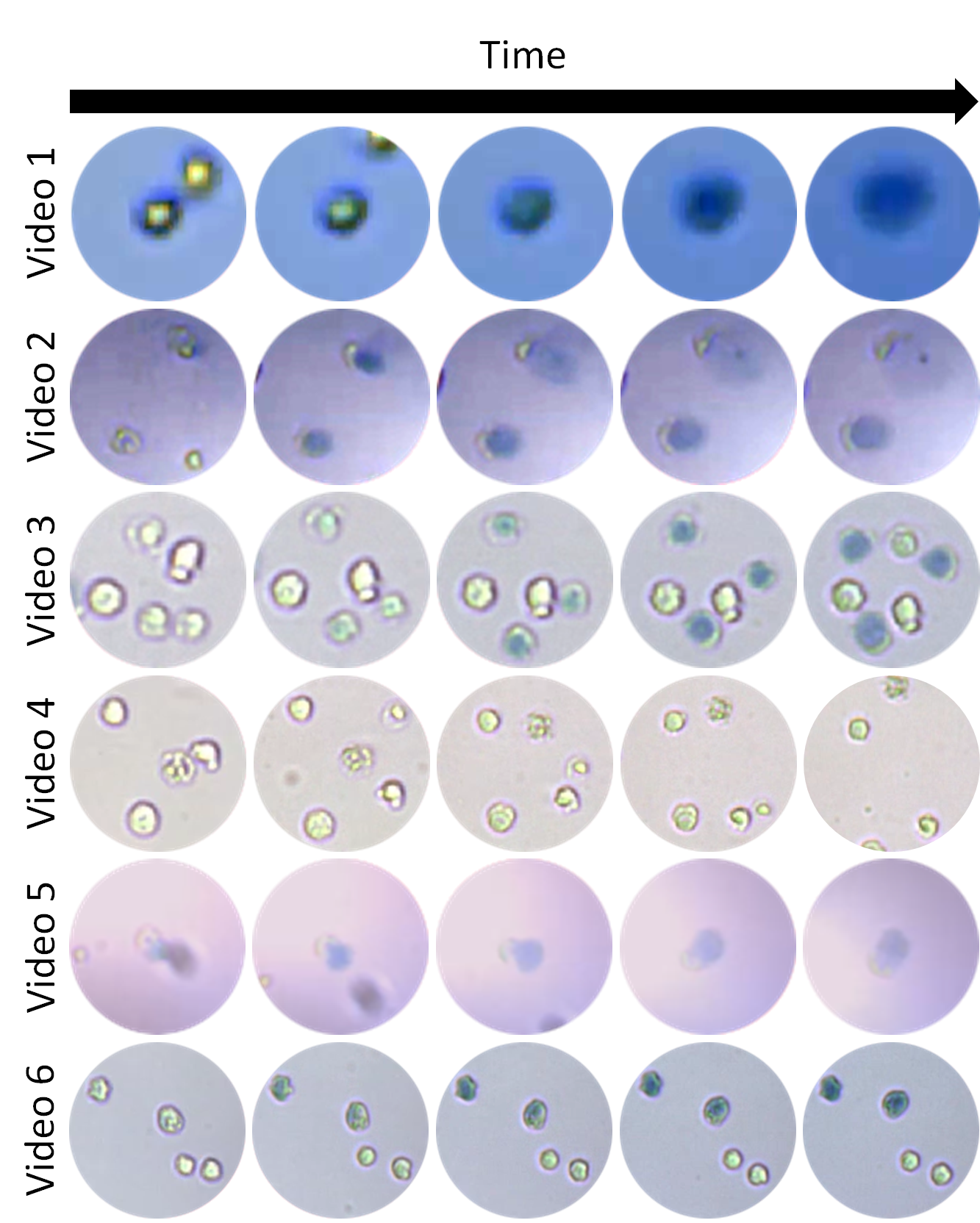

Supplement: S3 Fig — The progression of the images show the morphological changes to the cells staining with TB or PI. (TIF) [file pone.0227950.s009.tif]
